# Quantification of γδ T cells and HLA-DR+ NK cells does not predict emergence of new contrast enhancing lesions in MS patients suspending natalizumab treatment

**DOI:** 10.1371/journal.pone.0179095

**Published:** 2017-06-06

**Authors:** Mindaugas Paužuolis, Torsten Eich, Joachim Burman

**Affiliations:** 1 Biology Education Centre, Uppsala University, Uppsala, Sweden; 2 Department of Immunology, Genetics and Pathology, Uppsala University, Uppsala, Sweden; 3 Department of Neuroscience, Uppsala University, Uppsala, Sweden; Charite Universitatsmedizin Berlin, GERMANY

## Abstract

**Background:**

Natalizumab (NTZ) is a drug that has been widely used in the treatment of multiple sclerosis (MS). NTZ is very effective in suppressing inflammation, but if treatment is suspended many patients will experience relapses.

**Objective:**

To investigate if quantification of γδ T cells and HLA-DR^+^ NK cells could predict early disease reactivation after NTZ suspension.

**Methods:**

Absolute counts of γδ T cells and HLA-DR^+^ NK cells in whole blood were determined with flow cytometry in fifteen patients treated with NTZ. NTZ treatment was then withdrawn and patients were followed with clinical visits and MR investigations.

**Results:**

Patients with recurrent disease had higher absolute counts of γδ T cells 129 (±156) cells/μl in comparison to patients with stable disease 50.0 (±51.0) cells/μl but the difference was not statistically significant and largely driven by outliers. Patients with recurrent and stable disease had similar absolute counts of HLA-DR^+^ NK cells.

**Conclusion:**

Quantification of γδ T cells and HLA-DR^+^ NK cells could not predict active disease after NTZ suspension.

## Introduction

Multiple sclerosis (MS) is a severe neurological disorder leading to functional impairment and disability. At onset, most patients have a relapsing-remitting disease with periods of loss of function (relapses) followed by recovery. Such relapses are caused by localized inflammation in the brain and spinal cord, leading to disruption of neuronal signaling and tissue damage.

In the last two decades, several drugs have been developed to suppress and modulate inflammation in MS. Considerable effort has been put into proving the efficacy of these drugs, less so whether and when it is time to stop treatment. From natural history studies it is known that inflammation in MS tends to diminish with age [1]. Consequently, most patients will reach a point when disease-modifying drug (DMD) treatment is of lesser or no benefit. It is increasingly clear that first-line DMDs can be discontinued in many patients after a period of stability [2,3]. In other situations, withdrawal of treatment may have deleterious consequences. Discontinuation of the second line DMD natalizumab (NTZ) exposes patients to a high risk of severe relapses, if alternative treatment is not commenced [4].

NTZ is a monoclonal antibody that binds to α4β1 integrin on leukocyte plasma membranes, which prevents the interaction between α4β1 integrin and its cognate ligand vascular cell adhesion molecule-1 (VCAM-1). This blocking prevents extravasation of leukocytes to the effect that the leukocytes are trapped in the peripheral circulation and thus prevented from entering and damaging the central nervous system [5]. A serious drawback of this treatment strategy is the risk of progressive multifocal leukoencephalopathy (PML), which has been associated with long-term treatment. When this problem was first encountered [6], NTZ treatment was halted globally. Patients from the pivotal trials of NTZ were prospectively followed after treatment discontinuation. Return of disease activity ensued in many patients, mainly within 4–7 months [7]. This initial report has since then been followed by many others with similar results [8–11].

Since pathogenic lymphocytes are trapped within the peripheral circulation with NTZ treatment, it would be enticing to see whether it is possible to detect and quantify these in order to predict subsequent relapses after NTZ withdrawal. In a study made some years ago, a large number of immune subsets were investigated and related to disease activity [12]. The investigators could identify ten immune subsets that discriminated patients with high disease activity from patients without disease activity. Among these subsets were γδ T cells and HLA-DR^+^ NK cells, which can be readily quantified with standardized assays. We hypothesized that quantification of these in patients treated with NTZ would be able to predict subsequent relapses after discontinuation of natalizumab. To test this hypothesis we quantified γδ T cells and HLA-DR^+^ NK cells in blood samples from patients who were participating in a clinical trial investigating whether NTZ could be safely withdrawn after long-term treatment [4]. In order to understand if and how these subsets change with NTZ treatment we also quantified γδ T cells and HLA-DR^+^ NK cells from a group of patients commencing NTZ therapy.

## Material and methods

### Ethics approval

The study was approved by the Ethics Committee of the Medical Faculty of Uppsala University (DNr 2013/293). All subjects provided written informed consent.

### Power calculation

A power calculation was made based on based on data from Rinaldi et al [12]. We assumed an equal distribution of individuals with high and low disease activity. To reach a power of 0.90 with an α set to 0.05 seven individuals in each group was necessary for γδ T cells and ten individuals for HLA-DR^+^ NK cells. Therefore it was decided to include 20 patients in the study.

### Subjects

#### Patients initiating NTZ therapy

To investigate possible changes in the γδ T cell and HLA-DR^+^ NK cell subsets after initiation of NTZ therapy seven patients commencing NTZ treatment were included. Their characteristics are summarized in Table 1.

**Table 1:** Characteristics of the multiple sclerosis patients participating in the study.

	NTZ initiation cohort	NTZ suspension cohort
*n* (f/m)	7 (5/2)	15 (10/5)
Age (yrs)	37 (22–49)	50 (32–72)
Disease duration (yrs)	8 (2–16)	14 (6–37)
Age at start of NTZ treatment (yrs)	38 (23–49)	42 (26–65)
Age at suspension of NTZ (yrs)	n/a	50 (32–73)
Duration of NTZ treatment (yrs)	n/a	5 (4–8)
Previous treatment	IFN- β	IFN-β, glatiramer acetate, IVIg and mitoxantrone

Data are presented as medians with (range).

#### Patients suspending NTZ treatment

Patients who were participating in a clinical trial investigating whether NTZ could be safely withdrawn after long-term treatment [4] were recruited to the present study. In brief, the clinical trial contained patients who had been treated with natalizumab for at least five years with no clinical signs of disease activity. Their characteristics are summarized in Table 1. The first patient was included March 20^th^ 2014 and the last patient was included October 20^th^ 2014. Further recruitment to the study was halted for ethical reasons when a significant portion of the first 15 patients experienced severe relapses.

### Evaluation of disease activity

#### Patients initiating NTZ therapy

Patients commencing NTZ treatment were evaluated for disease activity at a follow-up visit at the end of the study (after six months).

#### Patients suspending NTZ treatment

Prior to entering the study, patients suspending NTZ were followed with scheduled clinical visits with EDSS scoring twice a year. Brain MRI was performed yearly. At baseline all patients had an MRI scan of the brain and spinal cord and MRI was repeated at three and six months. Patients were instructed to contact the study nurse if symptoms consistent with a relapse occurred. If so, an extra clinical visit was scheduled and an extra MRI was made.

Patients who had at least one gadolinium-enhancing lesion on any MRI scan performed at any time point after NTZ suspension were considered to have active disease.

### Blood samples

#### Patients initiating NTZ therapy

Ten ccs of venous blood were drawn from patients starting NTZ at baseline and then again three and six months after treatment commenced.

#### Patients suspending NTZ treatment

Ten ccs of venous blood were drawn bi-monthly starting at six months before suspension of NTZ treatment (at -6, -4, -2 and 0 months). After NTZ suspension further blood samples were taken at three and six months on the same day as the MRI scans. Some patients experienced severe relapses before follow-up at 6 months and started alternative treatment. Those patients did not leave blood samples at 6 months.

#### Quantification of γδ T cells and HLA-DR^+^ NK cells

The number and proportions of γδ T cells and HLA-DR^+^ NK cells was quantitated with flow cytometry in fresh whole blood samples. Measurements of absolute counts of T cells and NK cells were made with BD Truecount Tubes (Becton, Dickinson and Company, San Jose, CA). The antibodies used are detailed in Table 2. Matching isotype controls were used. γδ T cells were defined as CD3^+^ CD4^-^ CD8^-^ γ/δ TCR^+^ lymphocytes. HLA-DR^+^ NK cells were defined as CD3^-^ CD16^+^ CD56^+^ HLA-DR^+^ lymphocytes. The cells were acquired on a BD FACS Canto II. Flow cytometry data were analyzed with FlowJo v 10.1 (FlowJo LLC, Ashland, OR).

**Table 2:** Antibodies used for flow cytometry.

Antibody target	Fluorochrome	Clone	Manufacturer
CD3	PerCP	SK7	Becton Dickinson
CD4	FITC	SK3	Becton Dickinson
CD8	APC	SK1	Becton Dickinson
γ/δ TCR	PE	11F2	Becton Dickinson
CD16	PE	3G8	BD Pharmingen
CD56	PE	NCAM 16.2	Becton Dickinson
HLA-DR (L243)	PE-Cy7	L243	BD Pharmingen

### Statistical analysis

The statistical analysis was carried out with GraphPad Prism 6 (GraphPad Software, San Diego, CA). The Mann-Whitney test was used to establish statistical significance between two groups and the Friedman test was used to establish statistical significance between different time points within the same group. A p-value of 0.05 was considered to be statistically significant. Aggregated data is described as mean ± standard deviation (SD) or as median with range.

## Results

### Clinical outcomes

#### Patients initiating NTZ therapy

After the initiation of NTZ treatment, all patients were stable and did not experience clinical relapses or increase in disability.

#### Patients suspending NTZ treatment

Six out of fifteen patients had active disease with gadolinium-enhancing lesions on MRI within the first six months after suspending NTZ treatment and all of them also had a clinical relapse (Table 3).

**Table 3:** Summary of patients with gadolinium enhancing lesions after NTZ suspension.

Sex	Age at NTZ suspension	Clinical relapse	No of Gd+ lesions	EDSS change
F	55	YES	15	2.0 -> 3.5
F	31	YES	9	6.0 -> 6.0
M	50	YES	32	1.0 -> 2.0
F	58	YES	9	2.0 -> 6.0
F	50	YES	2	3.0 -> 3.5
F	52	YES	1	3.0 -> 5.0

### NTZ treatment increased the absolute numbers of γδ T cells and HLA-DR^+^ NK cells, but the relative proportions remained constant

**Fig 1:**
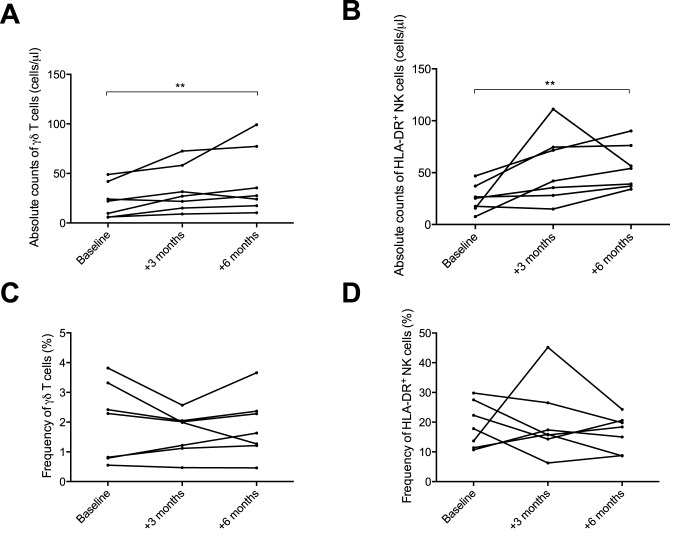
Quantification of γδ T cells and HLA-DR^+^ NK cells in MS patients commencing NTZ treatment.

As a first objective, we wanted to determine if and how γδ T cell and HLA-DR^+^ NK cell subsets change with NTZ treatment. After the introduction of NTZ there was a statistically significant increase in γδ T cell count (p = 0.0036) after 6 months of the treatment (Fig 1A). The average total number of γδ T cells increased from 22.6 (±17.3) cells/**μ**l at baseline to 41.6 (±33.4) cells/**μ**l 6 months after NTZ initiation. The proportion of γδ T cells in the total T cell population remained stable over the period of observation for most patients (Fig 1C).

Similarly, the HLA-DR^+^ NK cell counts increased from baseline to 6 months after NTZ initiation (p = 0.0036) (Fig 1B). The average HLA-DR^+^ NK cell counts increased from 25.3 (±13.3) cells/**μ**l at the baseline to 55.3 (±21.2) cells/**μ**l 6 months after NTZ initiation. The proportion of HLA-DR^+^ NK cells in the total NK cell population was fairly stable (see Fig 1D).

### γδ T cell and HLA-DR+ NK cell populations were stable during NTZ treatment

**Fig 2:**
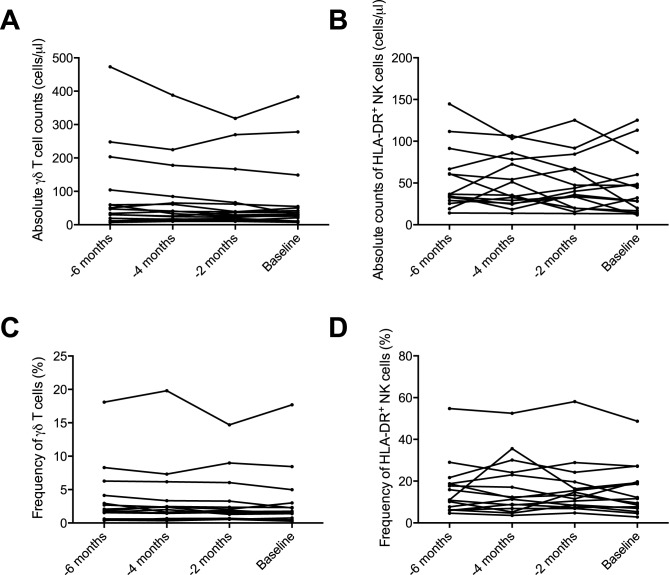
Quantification of γδ T cells and HLA-DR^+^ NK cells in MS patients before withdrawal of NTZ.

As a second objective, we wanted to investigate if the levels of γδ T cells and HLA-DR^+^ NK cells populations were stable in patients who had been treated with NTZ for a long time. The absolute counts of γδ T cells remained fairly constant over a period of six months (pooled CV 22.4, Fig 2A). Similarly, the proportions of γδ T cells in the total T cell population were stable (pooled CV 18.3, Fig 2C).

The absolute counts of HLA-DR^+^ NK cells (pooled CV 23.4, Fig 2B) and the proportions of HLA-DR^+^ NK cells in the total NK cell population (pooled CV 19.6, Fig 2D) were also stable during the observation period.

### After NTZ suspension absolute numbers of γδ T cells and HLA-DR^+^ NK cells approached the levels of untreated patients, but the relative proportions remained constant

**Fig 3:**
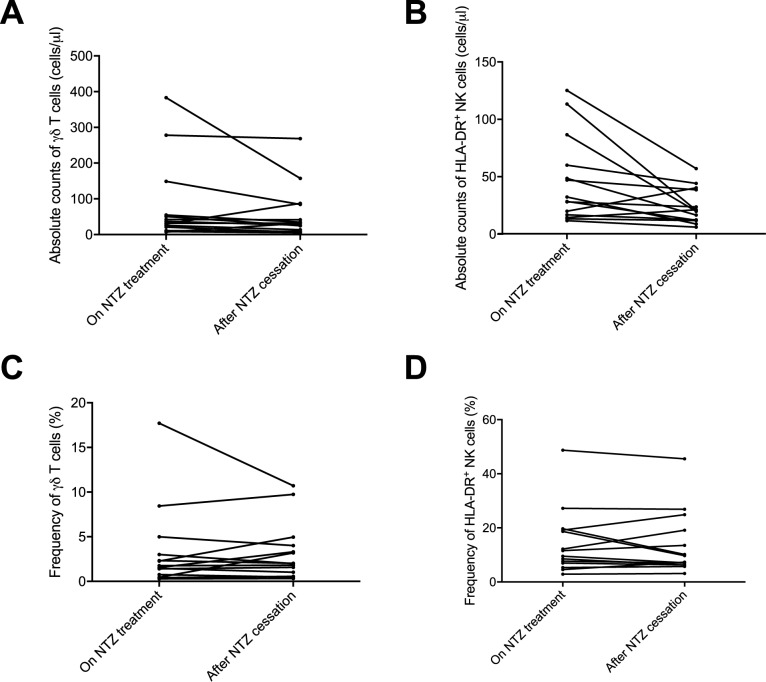
Quantification of γδ T cells and HLA-DR^+^ NK cells in MS patients before and after withdrawal of NTZ.

The absolute counts of γδ T cells decreased in a majority of patients after the cessation of NTZ (Fig 3A) and the average number of γδ T cells decreased from 80.7 (±113) cells/**μ**l at the baseline to 58.7 (±73.3) cells/**μ**l after the cessation of NTZ, but the difference was not statistically significant (p = 0.70). The proportion of γδ T cells in the total T cell population remained unchanged after the suspension of NTZ (Fig 3C).

The absolute counts of HLA-DR^+^ NK cells decreased after the termination of NTZ (Fig 3B). The average total number of HLA-DR^+^ NK cells decreased from 46.1 (±37.5) cells/**μ**l at baseline to 23.8 (±15.3) cells/μl after the suspension of NTZ, which was borderline statistically significant (p = 0.08). The proportion of HLA-DR^+^ NK cells in the total NK cell population remained unchanged (Fig 3D).

### Quantification of γδ T cells and HLA-DR+ NK cells could not predict relapses

**Fig 4:**
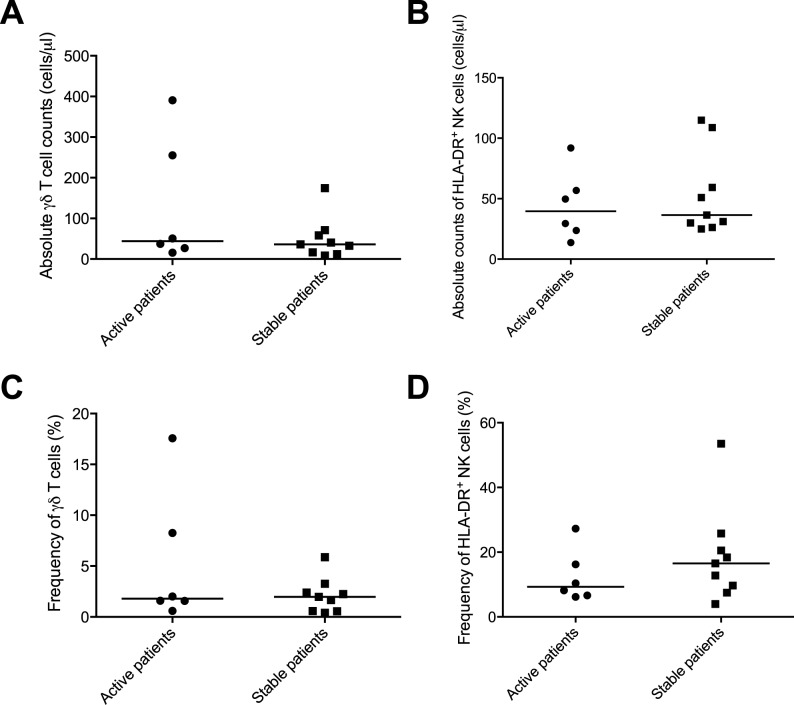
Quantification of γδ T cells and HLA-DR^+^ NK cells in MS patients with active and stable disease. Patients were considered to have active disease if they had any gadolinium enhancing lesions at any point during the follow-up period.

A numerical difference in the average absolute counts of γδ T cells between patients with recurrent disease and stable patients was observed; recurrent disease 129 (±156) cells/μl and stable disease 50.0 (±51.0) cells/μl which was not statistically significant and largely driven by outliers (Fig 4A). The same trend was observed in the proportion of γδ T cells in the total T cell population The mean proportion of γδ T cells was 5.27 (±6.64) % for patients with active disease and for stable patients 2.10 (±1.71) % (Fig 4C).

The absolute counts of HLA-DR^+^ NK cells were fairly similar in all patients (Fig 4B). Patients with recurrent disease had 44.2 (±28.4) cells/μl and stable patients 53.6 (±35.0) cells/μl of HLA-DR^+^ NK cells. The proportions were also similar, with 12.5 (±8.13) % for active patients and 18.7 (±14.7) % for stable patients (Fig 4D).

**Fig 5:**
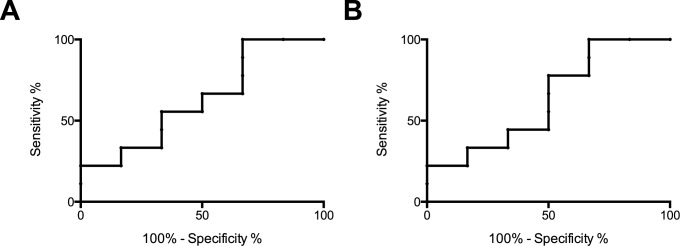
Predictive value of absolute counts of γδ T cells and HLA-DR^+^ NK cells. A) ROC curve illustrating sensitivity and specificity of the absolute counts of γδ T cells measured before NTZ withdrawal for prediction of subsequent development of gadolinium enhancing lesions. B) ROC curve illustrating sensitivity and specificity of the absolute counts of HLA-DR^+^ NK cells measured before NTZ withdrawal for prediction of subsequent development of gadolinium enhancing lesions.

To evaluate the predictive value of quantification of γδ T cells and HLA-DR^+^ NK cells for active disease after NTZ suspension we created ROC curves of the absolute counts, which clearly demonstrates that this test cannot reliably predict active disease (Fig 5).

## Discussion

From a clinical point of view, it would be very useful to have access to a tool that could predict recurrent disease activity after NTZ suspension, which is not uncommon in the face of pregnancy or development of a high JC-virus index [13]. The main objective of this study was to investigate whether high levels of γδ T cells and/or HLA-DR^+^ NK could predict future relapses in MS patients suspending NTZ treatment. We could find no support for this hypothesis. However, this does not exclude that there may exist biologically relevant differences between active and stable patients in these immune subsets.

In the first part of the study, we could observe an increase in the absolute counts of γδ T cells and HLA-DR^+^ NK cells 6 months after initiation of treatment, whereas the proportion of γδ T cells and HLA-DR^+^ NK cells did not change. This observation is consistent with previous reports that NTZ treatment leads to an overall increase in T cell populations and noticeable changes in the cytotoxic CD3^-^CD56^dim^ NK cell subset [14–16]. Similar to our study, the proportion of HLA-DR^+^ NK cells in the total NK cell population was unchanged [15]. While on treatment, the levels of γδ T cells and HLA-DR^+^ NK were fairly stable in individual patients. When NTZ treatment was suspended, we saw a decrease in the absolute counts γδ T cells and HLA-DR^+^ NK cells. Again, the proportion of γδ T cells and HLA-DR^+^ NK cells in the total T and NK cell populations was essentially unchanged.

In the second part of the study, we tried to determine if it is possible to predict who will get active disease after withdrawal of NTZ treatment. This part of the study was largely based on the results of Rinaldi et al. [12]. The validity of the hypothesis hinges on that the immune subsets of γδ T cells and HLA-DR^+^ NK cells are not differently affected by NTZ in individual patients. As demonstrated in the first part of the study the absolute counts were increased but the proportions were stable in individual patients. This suggests that our approach was feasible. We observed very similar frequencies of γδ T cells in active vs stable patients as Rinaldi et al. (active patients: 5.27 vs 5.5; stable patients 2.10 vs 2.3), suggesting that there may be a biologically relevant difference between active and stable patients in this immune subset. However, the interindividual variation of this metric was too high to be useful as a clinical test.

We could not confirm the findings of Rinaldi et al. in the frequencies of HLA-DR^+^ NK cells (active patients: 12.5 vs 18.4; stable patients 18.7 vs 9.2). This discrepancy may be due to differences in methodology; γδ T cells constitute an easily identified subpopulation of T cells and their quantification is very straightforward. In contrast, the expression of HLA-DR on NK cells is gradual and the classification of NK cells as HLA-DR^+^ or HLA-DR^-^ is therefore more difficult and somewhat subjective. Another possible explanation for the disparate findings is that NTZ affects NK cells in a hitherto unknown manner, which is different from the effect on T cells. Underlying differences in the studied cohorts, such as age or previous exposure to immunotherapy, may also have influenced the results.

Other strategies to measure immunological disease activity may be more successful. In a recent study it was suggested that a low frequency of Th17 cells in blood could act as a marker for MS relapse after the discontinuation of NTZ [17]. Patients under NTZ treatment had a higher proportion of Th17 cells in peripheral blood in comparison to healthy controls and MS patients with no history of NTZ treatment. After NTZ withdrawal the levels of Th17 cells decreased substantially in patients who subsequently experienced a relapse. Thus, quantification of Th17 cells may be a better way to predict future relapses after NTZ suspension with the drawbacks that NTZ was already discontinued when the measurements were made. Another study suggested that low level of NTZ saturation on T cells might predict biological relapses [18]. Patients who experienced early relapses after the discontinuation of NTZ had a low level of NTZ saturation on T cells in comparison with stable patients. Unfortunately, the study had very small sample size and was not statistically powered to evaluate NTZ saturation as a clinical test. Furthermore, the measurements of NTZ saturation were carried out after the withdrawal of NTZ limiting the usefulness of the test. Nevertheless, measurement of NTZ saturation may be useful to determine optimal wash-out times when transitioning from NTZ to another DMD.

## Conclusion

In summary, we could confirm the expected findings that the absolute counts of γδ T cells and HLA-DR^+^ NK cells increase with NTZ treatment, that the levels are fairly stable during treatment and that they decrease when NTZ treatment is withdrawn. Furthermore, we could demonstrate that the proportion of γδ T cells and HLA-DR^+^ NK cells are essentially unchanged with NTZ treatment. However, the main objective was negative and we conclude that the quantification of γδ T cells and HLA-DR^+^ NK cells does not predict active disease after NTZ suspension.

## Supporting information

S1 File Data file of measurements of γδ T cells. (XLSX)

S2 File Data file of measurements of HLA-DR^+^ NK cells. (XLSX)
